# Asymptomatic malaria and predictors among migrant farmworkers East Shewa zone Oromia Ethiopia

**DOI:** 10.1038/s41598-024-65470-x

**Published:** 2024-07-13

**Authors:** Gudeta Legesse, Weynshet Tafesse, Dagaga Kenea, Bereket Wake Subussa, Gezahegn Solomon Alemayehu, Tadesse Kebede, Lemu Golassa, Musa Mohammed Ali, Asrat Hailu

**Affiliations:** 1 Department of Medical Laboratory Science, College of Health Science, Arsi University, Asella, Ethiopia; 2 Department of Medical Laboratory Science, College of Medicine and Health Sciences, Wachamo University Hossana, Hosaina, Ethiopia; 3 College of Health Science, Defense University, Bishoftu, Oromia State Ethiopia; 4 Aklilu Lemma Institute of Pathobiology, Addis Ababa University, Addis Ababa, Ethiopia; 5 Department of Microbiology, Immunology, and Parasitology, College of Health Science, Addis Ababa University, Addis Ababa, Ethiopia; 6 School of Medical Laboratory Science, College of Medicine and Health Sciences, Hawassa University, Hawassa, Ethiopia

**Keywords:** Plasmodium, Asymptomatic malaria, Gametocyte, East Shewa, Ethiopia, Microbiology, Health occupations

## Abstract

Asymptomatic malaria can impact existing malaria control and elimination efforts around the world, particularly in Africa, where the majority of malaria cases and death occurs. This is a cross-sectional study aimed to determine the prevalence and predictors of asymptomatic malaria among migrant farmworkers from June to July 2020 in the Upper Awash Agro-industry, East Shewa zone, Oromia Regional State, Ethiopia. A total of 254 migrant farmworkers without signs and symptoms of malaria were enrolled. Data on socio-demographic characteristics and malaria prevention practices were obtained through a structured questionnaire. Venous blood samples were collected and diagnosed using microscopy, rapid diagnostic tests, and polymerase chain reaction (PCR). Data were coded, entered, and analyzed using SPSS version-21 statistical software. Multivariable logistic regression was used to assess associated factors. A *p* < 0.05 was considered statistically significant. The overall prevalence of asymptomatic malaria among farmworkers in this study was 5.1% [95% CI 1.6, 6.7]. The proportions of *Plasmodium falciparum* was 90.0% (9/10) while it was 10.0% (1/10) for *Plasmodium vivax*. Out of the microscopy and/or RDT-confirmed malaria cases, (n = 9; 100%) were confirmed to be *P. falciparum* by nested PCR, while (n = 3/122; 2.46%) were found to be *P. falciparum* among 50% negative cases with the microscopy and/or RDT. The gametocyte stage was detected in 40% of microscopically positive cases out of which 44.4% belongs to *P. falciparum*. Home area/origin of migrant laborers [AOR = 6.08, (95% CI 1.08, 34.66)], family history of malaria [AOR = 8.15, (95% CI 1.43, 46.44)], and outdoor sleeping [AOR = 10.14, (95% CI 1.15, 89.14)] were significantly associated with asymptomatic malaria. In conclusion, asymptomatic malaria was detected among farmworkers in the study area and it was significantly associated with outdoor sleeping, home area, and family history of malaria. Prevention tools and control strategies, particularly focusing on migrant farmworkers, should be considered to support the ongoing malaria control and elimination effort in Ethiopia.

## Introduction

Malaria is caused by Protozoan parasites of the genus *Plasmodium* that infect several species of vertebrates; five are known to infect humans, namely *Plasmodium falciparum, Plasmodium vivax, Plasmodium ovale, Plasmodium malariae,* and *Plasmodium knowlesi*^1,2^.

According to the World Health Organization (WHO) 2020 report, malaria remains the main public health problem; in 2019 there were an estimated 229 million cases of malaria worldwide in 87 malaria-endemic countries. The majority of malaria cases in 2019 occurred in the WHO African region, with an estimated 215 million cases in 2018, accounting for around 94% of cases. Likewise, 409,000 malaria death in 2019 were estimated in the world. Most of these deaths occurred in the WHO African region (94%)^3^.

According to the malaria risk stratification map in Ethiopia (FMOH, 2020), most Districts/Regions are in the category of low malaria transmission (annual incidence of parasites (API) = 5–9 cases/1000 population/year), while few Districts/Regions are in the high transmission area (API =  ≥ 50 cases/1000 population/year). In line with this, understanding the epidemiology of malaria in different transmission settings is important in designing an appropriate malaria intervention. In a low transmission setting, people infected with malaria will be symptomatic with a low level of parasitaemia due to a lower level of acquired immunity. In high transmission settings, the majority of the population will be asymptomatic due to the development of antimalarial immunity as a result of frequent exposure to the malaria parasite^4,5^.

Despite current efforts to control malaria in Ethiopia, the situation has not improved mainly due to increasing problems of resistance of parasites to relatively cheaper antimalarial drugs, the resistance of vectors to insecticides, low coverage of services prevention of malaria, poor access to medical care, the rudimentary infrastructure of health services, large population movements, limited human and financial resources, and the presence of asymptomatic malaria^6^.

Asymptomatic infections can be associated with high levels of gametocytes and probably serve as an important parasite reservoir, and have a significant contribution to maintaining the parasite for transmission^7^. Continuous exposures to *Plasmodium* parasites produce partial immunity in high transmission areas^8^, and also create an asymptomatic carrier state to play a role in determining malaria transmission in a given population^9^. Asymptomatic malaria patients play a critical role in the concept of a malaria elimination program. Current guidelines aim to improve the early detection of new symptomatic and subacute casesthrough the use of village health workers however; the strategy does not reach people with chronic low-density infections^10^.

Migrant workers often work during the cooler evening hours when there is a high rate of vector bites and often sleep in open fields or temporary shelters where vector control measures are not applied, exposing themselves to the sting of a mosquito. They often become infected while working and sleeping in agricultural fields^11,12^.

Asymptomatic malaria can be detected both in stable endemic regions and in areas of unstable transmission. However, much attention has been paid to symptomatic malaria infections and relatively little attention to asymptomatic malaria. Because the asymptomatic host serves as a reservoir for the malaria parasite, it is now recognized as a major obstacle to malaria elimination and a new challenge for a national strategic plan for malaria prevention and control. Therefore, knowledge of the baseline prevalence of malaria among migrant farmworkers is of utmost importance in guiding and scaling up malaria control intervention programs. To the best of our knowledge and belief, there is no documented study conducted in Upper Awash Agro-industry. Therefore, the present study was designed to determine the prevalence and predictors of asymptomatic malaria in those who harbor the parasites in the farmworker community of Upper Awash Agro-industry, East Shewa, and Southeast Ethiopia.

## Materials and methods

### Study area

A community-based cross-sectional study was conducted to determine the prevalence and predictors of asymptomatic malaria parasites among Upper Awash Agro-industry migrant farmworkers in Boset District, East Shewa, South-eastern Ethiopia; from June to July 2020. Boset District is located in southeastern Oromia State; it lies in a tropical climatic Zone with an altitude of 1100 to 2700 m above sea level, an average annual temperature that varies between 25 and 30 °C for the tropical (Kolla) and between 15 and 20 °C for the subtropical (woinadega) seasons. The total population of Boset District for the year 2017 was projected at 189,795 of which 42,793 (22.5%) are urban population and 147,002 (77.5%) are rural population^13^. In terms of the drainage system, the district falls into the Awash River basin, with no other major streams and lakes. It is an agricultural area where extensive agriculture is carried out in Ethiopia through the irrigation of the Awash River. This area is known to be malarious with intense transmission patterns.

### Study population

All farmworkers who live in Nura Hera of Upper Awash Agro-industry camps, all migrant farmworkers with no disease symptoms/signs of malaria in the last four days, axillary temperature ≤ 37 °C, those willing to participate in the study, who sign the informed consent and who lived for 6 months and above in Nura Hera camps of Upper Awash Agro-industry migrant farmworkers.

### Sample size determination and sampling technique

The sample size was determined using a single population proportion formula using the following assumptions; p = 18.4% prevalence of asymptomatic malaria among migrant workers from the study conducted in northwestern Ethiopia, Armachiho^14^, Zα/2 = 1.96 at a 95% confidence interval (CI), d = 5% margin of error and n = sample size. Taking a non-response rate of 10%, the total sample size (n) was 254.

### Sampling techniques

**Figure 1:**
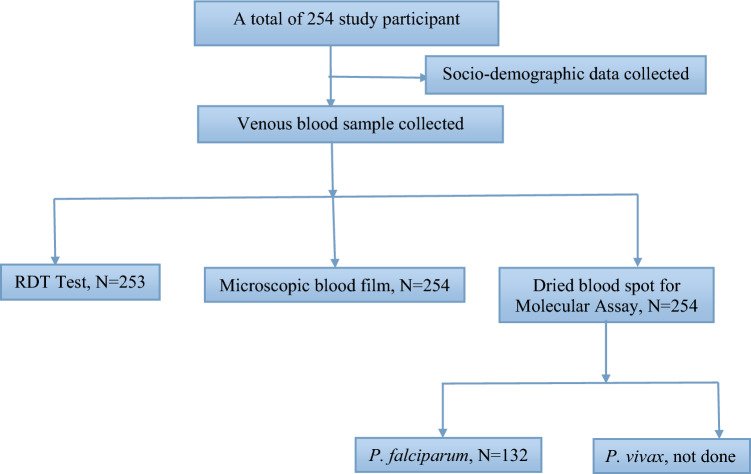
Study flow chart for malaria RDT, Microscopy, and Molecular assay.

A multi-stage sampling technique was used to select the representative sample size. Nura Hera of Upper Awash Agro-industry has 13 camps. Six camps were selected by using the lottery method. To determine the proportional sample size for each selected camp, the recent registration lists on households were used. Based on the number of households, the estimated sample size was proportionally distributed to the selected six camps; for camp 5th: 44, for camp 6th: 51, for camp monopol: 37, for camp 7th: 40, for camp 8th: 31, and for camp 10th: 51 households (Fig. 1). Each household was selected using a systematic random sampling technique. Finally, one individual within selected households above 18 years old were selected using the lottery method.

### Socio-demographic data

A pre-structured questionnaire was used to collect socio-demographic data such as gender, age, marital status, family size, and educational level by interview administered method.

### Blood sample collection and processing

Approximately 4 ml of venous blood samples were collected aseptically using a sterile disposable syringe for thick and thin blood smears for microscopy, RDT, and dried blood spot on Whatman filter paper and transported in a zip bag with desiccant to Aklilu Lemma Institute of pathobiology, Addis Ababa University for PCR assay. All participants were tested for malaria first using RDT and then confirmed by microscopy. For some of them we have diagnosed malaria using Polymerase Chain Reaction (PCR) method.

### Examination of blood films

For each participant, two blood films (thick and thin blood smears on the same slide) were prepared from a venous blood sample. Thin blood smears were fixed with methanol for 30 s and stained with a 3% Giemsa solution for 30 min after air drying. Following standard protocols, the stained smears were investigated with a light microscope with a high-power (100x) magnification objective to detect malaria parasites. The thick smear preparations were first examined with a high magnification objective (100 ×) for the presence of parasites. Consequently, when the slide was found to be positive, the *Plasmodium* species were identified by examining the thin blood smears under the 100 × objective. The identification of the species was carried out based on the shape of the ring stage, the size of the infected red blood cells, the shape of the gametocytes, the presence of chromatin dots, the number of merozoites in the schizont, and others. Results were qualitatively classified as negative (no malaria parasites observed), positive for specific *Plasmodium* species, or mixed infection. At least 100 high power fields (100 × objective) were examined before reporting a negative result^15^. The asexual density of the *Plasmodium* parasite per microliter (μl) of blood was determined by counting the number of parasites per 200 white blood cells in a thick blood smear assuming a standard total leukocyte count of 8000/μl. The degree of parasite density was classified as mild, moderate, and severe when the counts were between 1 and 999 parasites/µl, 1000–9999/µl, and > 10,000/µl, respectively, following the method described elsewhere^16^. Gametocyte density was quantified against 500 leukocytes. This was also converted to the number of gametocytes per microliter of blood, assuming a standard approximation of a leukocyte count of 8000/μl.

### Rapid diagnostic tests

The SD BIOLINE Malaria Ag P.f / P.v POCT Test Kit (Standard diagnostic, Inc, Germany, Lot No. 145021) was used to capture malaria antigens and was performed according to the manufacturer’s instructions. The kit targeted *P. falciparum*-specific HRP-2 malaria antigens and *P. vivax*-specific *Plasmodium* lactate dehydrogenase (pLDH) antigens. The RDT test results were read after 20 min and interpreted as follows: Two bands—one band in the control area and another in the *Plasmodum falciparum* (Pf) area (Pf test line) indicating *P. falciparum* infections; Two bands- one band in the control area and another band in the *Plasmodum vivax* (Pv) area (Pv test line) indicate a positive result for *P. vivax*; Three bands- the bands in the control area, the Pv area, and the Pf area indicate a mixed infection; Only one band in the control area in the result window shows a negative result and In case the control line does not appear, the result is interpreted as invalid.

### DNA extraction and PCR amplification

A nested amplification of the *Plasmodium* 18S rRNA gene was performed to identify positive infection and parasite species using published protocols^17,18^. The parasite DNA was extracted using the Chelex method^19^. PCR was performed in all positive cases by microscopy and/or RDT and for fifty percent of the negative cases. *Plasmodium* species were identified by microscopy and RDT, and *P. falciparum* was confirmed by *P. falciparum*-specific primers.

### Data quality management

The questionnaire was assessed by conducting a pretest before the data collection period. The training was conducted for the research team before data collection. Before data entry, the returned questionnaires were verified for completeness and corrective action was taken. To ensure maximum participation, households with absences were screened for a second time the following day to recruit those missing on the first visit. All laboratory supplies, such as rapid test kits, slides, thermometers, EDTA tubes, and sample transport systems, were reviewed by experienced laboratory professionals. Samples were also reviewed for serial numbers, quality, and collection procedures. Laboratory professionals involved in RDT, light microscopy examination, and PCR were trained in malaria diagnosis and quality assurance training. Additionally, to minimize missing parasite identification and discrepancy, each microscopic slide was examined by two trained professionals at the Arsi University School of Health Sciences medical laboratory center. Rapid test kit verified to have an expiration date, correct collection procedures, and samples, as well as built-in control appearances. Inconsistent light microscopy results were reviewed again to confirm the findings.

### Quality control

All test procedures and interpretation of results were performed using standard operating procedures (SOPs). The expiration date of the reagents, materials, CareStartTM Malaria HRP2/pLDH (Pf/Pv) Combo (RDT), and PCR reagents and readers were checked daily before data collection was performed. The quality of the Giemsa staining was evaluated by positive and negative control of blood smears. All positive and 5% negative microscope slides were re-examined by a senior medical laboratory technologist who has a master’s degree in medical parasitology; Arsi University with more than 3 years of experience in the diagnosis of malaria in the Hospital who did not know the first data set. There was no discordant result between the first and the second examiner.

### Data processing and analysis

Data were coded, entered into Epi data version 3.1, cleaned, transported to SPSS, and analyzed using the Statistical Package for Social Sciences (SPSS) version 21. Parasitaemia density was expressed as the number of asexual *Plasmodium* per microliter of blood, assuming a leukocyte count of 8000 per microliter. Frequency distributions and proportions were calculated and the association between dependent and independent variables was explored using bivariate and multivariate logistic regression. Those variables associated with the outcome variable in the bivariate regression model *P*-value < 0.25 were entered into multivariate analysis to control possible confounders. Prevalence figures were calculated for the total study population. A *p*-value less than 0.05 was considered statistically significant. Finally, the data were described using text, frequency, odds ratio, and percentages.

### Ethical approval

The study was approved by the Ethical Review and Research Committee of the Department of Medical Microbiology, Immunology and Parasitology, College of Health Sciences, Addis Ababa University. Permission to conduct the study was obtained from the Regional Health Office, the Zonal Health Office, the District Health Office, and the Upper Awash Agro-industry Central Office. Study subjects were informed about the aim and objectives of the study in the local language. Written informed consent was obtained from the participants (age ≥ 18 years). For participants who are unable to read and write informed consent was obtained from their guardian or legally authorized representatives. Once data was collected from each study participant and laboratory test results were kept confidential by identifying patient information. Positive results with parasitic infection were timely forwarded to study participants and referred to the nearest health institution for appropriate treatment. All procedures were carried out according to declaration of Helsinki.

## Results

### Socio-demographic characteristics

A total of 254 farmworkers selected from 6 farm sites were included in this study. The majority were male (n = 149; 58.4%) and their mean age was 31.7 (range 18–72 years) with a standard deviation of 12.2. Most of the participants were in the age group 18 to 29 years (n = 125; 49.2%). Of the total study participants, 91(35.7%) could not read or write. Most of the participants (n = 209; 82.4%) were married. The mean family size in the present study was 2.7 (SD ± 2.12) with a range of 1 to 9 people per household. Of the total study participants (n = 152; 59.8%) were from the highland area (Table 1).

**Table 1:** Socio-demographic characteristics of the study participants, Upper Awash Agro-industry migrant farmworkers; Southeast Ethiopia, June to July 2020 (N = 254).

Variables	Category	Frequency (%)
Gender	Male	152 (59.8%)
	Female	102 (40.2%)
Age group	18–29	127 (50.0%)
	30–44	78 (30.7%)
	≥ 45	49 (19.3%)
Marital status	Married	209 (82.3%)
	Single	43 (16.9%)
Family size	≤ 3	177 (69.7%)
	6-Apr	63 (24.8%)
	≥ 7	14 (5.5%)
Educational level	Unable to read and write	91 (35.8%)
	Read and write	16 (6.3%)
	Elementary school	87 (34.3%)
	High school and above	60 (23.6%)
Origin/home area	Highland	152 (59.8%)
	Lowland	102 (40.2%)
Frequency of visiting the sites	Once	187 (73.6%)
	Twice	43 (16.9%)
	Third	24 (9.4%)

### Prevalence of asymptomatic malaria

The overall prevalence of asymptomatic malaria among farmworkers was 5.1% (13/254) 95% CI [1.6, 6.7], with two species of *Plasmodium* identified. The relative proportions of *Plasmodium* species among positive cases were 90.0% (9/10) *P. falciparum*, and 10.0% (1/10) *P. vivax* infections. No mixed infection was detected. PCR test was done for all samples which were tested positive for *Plasmodium* species by microscopy and//or RDT, and 50% negative cases. Accordingly, all 100% (9/9) cases of *P. falciparum* were confirmed by nested PCR. Among 122 randomly chosen asymptomatic microscopy and/or RDT negative cases, 2.46% (3/122) *P. falciparum* was detected for *P. falciparum* by PCR (Table 2).

**Table 2:** The prevalence of asymptomatic malaria as determined by microscopy, RDT, and PCR; Upper Awash Agro-industry migrant farmworkers, Southeast Ethiopia, June to July 2020 (n = 254).

Individuals	Microscopy		RDT		PCR		Total
	Pos n (%)	Total	Pos n (%)	Total	Pos n (%)	Total	Pos n (%)
Asymptomatic malaria	10 (3.9)	254	10 (3.9)	254	12(4.7%)	254	12 (4.7%)
*P. falciparum*	9 (90%)	10	9 (90%)	10	12 (92.3%)	13	12 (92.3%)
*P. vivax*	1 (10%)	10	1 (10%)	10	Not done		

RDT: Rapid Diagnostic Test, Pos: Positive; PCR: Polymerase chain reaction.

The gametocyte stage was detected in 40% (4/10) of microscopically positive cases and 44.4% (4/9) of *P. falciparum-*positive gametocytes; however, gametocytes of *P. vivax* were not detected. Of the 10 diagnosed with asymptomatic *Plasmodium* infection; 8(80.0%) and 2(20.0%) had mild and moderate parasitemia, respectively.

### Prevalence of asymptomatic malaria by Sex and age

Among the participants, 5.9% (9/152) were males and 3.9% (4/102) were females. Based on the age-specific prevalence, the highest prevalence of asymptomatic malaria was observed among the age group of 18–29 years. The overall prevalence of asymptomatic malaria was 5.1% (13/254) in Upper Awash Agro-industry farmworkers (Table 3).

**Table 3:** The distribution of asymptomatic malaria by gender and age groups by Light Microscopy, RDT and PCR, Upper Awash Agro-industry migrant farmworkers, Southeast Ethiopia, June to July 2020 (n = 254).

Variables	Upper Awash Agro-industry farmworkers	
	Pos n (%)	Total
Gender		
Male	9(5.9%)	152
Female	4 (3.9%)	102
Age group (year)		
18–29	8 (6.3%)	127
30–44	4 (5.1%)	78
≥ 45	1 (2.0%)	49
Total	13 (5.1%)	254

### Associated factors of malaria infection

Possible factors associated with malaria that showed a *p* < 0.25 in the bivariate analysis were selected and entered for multivariable logistic regression to control for confounding factors. Variables such as gender, age group, family size, ITN availability, a ratio of ITN to family size, ITN use, presence of eaves in the house, presence of any holes in the wall, outdoor activities overnight, family history of malaria, and previous malaria infections were selected and entered into the backward stepped multivariable logistic regression model. Risk factor evaluation generally showed that home area/origin, outdoor activities at night, and family history of malaria were significantly associated with asymptomatic *Plasmodium* infection (*p* < 0.05), while gender, age group, bed net usage, IRS coverage, family size, presence of eaves, presence of holes on the walls of a house, were not significantly associated with asymptomatic *Plasmodium* infection (*p* > 0.05) (Table 4).

**Table 4:** Bivariate and multivariable logistic regression analysis of associated factors for asymptomatic malaria; Upper Awash Agro-industry migrant farmworkers, Southeast Ethiopia, June to July 2020 (n = 254).

Variable	Category	n (%)	Malaria		COR (95% CI)	*p*-value	AOR (95%CI)	*p*-value
			Positive	Negative				
Gender	Male	152 (59.8%)	9 (5.9%)	143 (94.1%)	0.36 (0.055, 1.731)	0.202	2.899 (0.502, 16.756)	0.202
	Female	102 (40.2%)	4 (3.9%)	98 (98.1%)	1		1	
Origin/home area	Highland	134 (59.8%)	11 (8.2%)	123 (91.8%)	0.246 (0.051, 1.82)	0.080	6.08 (1.067, 34.664)	0.042
	Lowland	120 (40.2%)	2 (1.7%)	118 (98.3%)	1		1	
Utilization of ITN	Yes	218 (85.8%)	11 (5.05%)	207 (94.95%)	1		1	
	No	36 (14.2%)	2 (5.6%)	34 (94.4%)	2.48 (0.61, 10.02)	0.204	0.29 (0.056, 1.488)	0.138
Outdoor Sleeping	Yes	90 (35.4%)	10 (11.1%)	80 (88.9%)	0.101 (0.013, 0.897)	0.031	10.14 (1.145, 89.1430	0.037
	No	164 (64.6%)	3 (1.8%)	161 (98.2%)	1		1	
History of malaria infection	Yes	176 (69.3%)	7 (3.98%)	169 (96.02%)	1		1	
	No	78 (30.7%)	6 (7.7%)	72(92.3%)	2.34 (0.658, 8.331)	0.189	0.34 (0.083, 1.40)	0.135
Family history of malaria infection	Yes	113 (44.5%)	10 (8.8%)	103 (91.2%)	103 (91.2%)	0.37	8.15 (1.43, 46.44)	0.018
	No	141 (55.5%)	3 (2.1%)	138 (97.9%)	138 (97.9%)		1	

AOR, adjusted odds ratio, COR, Crude odds ratio, OR, odds ratio.

Individuals from the highlands were approximately six times more likely to get malaria than those from the lowlands [AOR = 6.08, (95% CI 1.08, 34.66)]. Individuals who had experience with outdoor activities at night were 10 times more likely to have asymptomatic malaria infection than their counterparts [AOR = 10.14, (95% CI 1.15, 89.14)]. Individuals with a family history of malaria were 8 times [AOR = 8.15, (95% CI 1.43, 46.44)] more likely to have an asymptomatic *Plasmodium* infection than those without a family history of malaria (Table 4).

## Discussion

The detection and quantification of reservoirs of the malaria parasite in a given area in asymptomatic individuals are important for the reduction of transmission and elimination of malaria. However, the diagnosis of asymptomatic malaria is becoming a challenge due to low levels of parasitemia and the absence of more sensitive diagnostic tools^20^.

The present study provides important information on the magnitude of asymptomatic malaria and the associated risk factors among farm workers in the Upper Awash Agro-industry in the East Shewa Zone, where malaria transmission is markedly seasonal and unstable.

In this study, the overall prevalence of asymptomatic *Plasmodium* infection among migrant farmworkers was 5.1% by both microscopy and/or RDT, and PCR. Surprisingly, no case of mixed infection was found. This finding is in line with a study conducted in three regions (Oromia, Amhara, and SNNP) of Ethiopia 4.1%^21^, 4.2% in Debre Elias District, North West Ethiopia^22^, 5% in West Arsi Zone, Southeast Ethiopia^23^ and 4.3% in India^24^.

Overall, the 5.1% prevalence of asymptomatic malaria among migrant farmworkers in the Upper Awash Agro-industry as determined by microscopy, RDT, and PCR was substantially lower than in studies conducted in Gondar Zuria District, Northwest Ethiopia 12%^25^, 18.4% West Armachiho, Northwest Ethiopia^14^, 16% in Dilla town, Ethiopia, ^26^ 36.3% in Tanzania^27^, 20% in 2013 and 22% in 2014 in India^28^, 12% in Gabon^29^, 12.6% in Kenya^30^, 39.2% in Mozambique^31^, 35% in Senegal^32^, and 32% in the Gambia^33^. Possible reasons for the difference could be differences in the intensity of malaria transmission, study design, geographic location (precipitation, temperature, and elevation), the quality of house, nature of population, sample size, study period, and malaria control program applied in the study area.

The study conducted in Mirab Abaya District, Southern Ethiopia^34^, China-Myanmar Border^35^, Myanmar^36^, and Bangladesh^37^ showed that the prevalence of asymptomatic malaria was 1.2, 0.3, 1.44, and 1% respectively which are lower than our findings. The reason for this discrepancy might be due to differences in the population such as genetics, epidemiology of malaria, study design, sample size, geographical location, test methods, and sampling techniques.

In this study, the predominance of *P. falciparum* (90.0%) over *P. vivax* infections (10.0%) was demonstrated. This finding is comparable with studies conducted in Pawe^38^, Mirab Abaya^34^, Debre Elias^22^, and Sanja^39^, which showed a predominance of *P. falciparum*. In contrast, the study conducted in Gondar Zuria, Northwest Ethiopia^25^, East Shewa Zone; Ethiopia^40,41^ showed that *P. vivax* is predominant over *P. falciparum.* The difference may be a result of climate variation among study areas which affects the life cycle of the parasite, parasite adaptation among *P. vivax* and *P. falciparum*, variety in the epidemiological distribution of *Plasmodium* species in a different part of Ethiopia, and the presence of relapse for *P. vivax.*

The prevalence of malaria was higher in men than in women, but it was not statistically significant. This finding is supported by previous studies conducted in Ethiopia^39,42^ and in other parts of the world^35,43^. This could be because men are more active in outdoor activities than women, while women spend most of their time at home; this puts men more exposed to outdoor activities and faces a higher risk of mosquito bites.

In this study, a PCR test was done for all samples which were tested positive for *Plasmodium* species by microscopy and/or RDT, and 50% negative cases. Among 122 randomly chosen asymptomatic microscopy and/or RDT negative cases, 2.46% (3/122) cases were detected for *P. falciparum* by PCR, suggesting that asymptomatic parasitemia in these samples could be below the threshold of microscopy and RDT. This is in line with a study done in South-central Oromia, Ethiopia^23^. Because PCR can detect parasitemia as low as 0.02 parasites per µl, it is not surprising to note the superiority of PCR over other classical diagnostic methods^44^.

The use of long-lasting insecticide-treated nets is an effective form of protection that reduces mosquito bites and thus reduces the risk of malaria, severe disease, and death in malaria-endemic regions^30^. In this study, the use of ITNs is not associated with positive cases. Educating migrant farmworkers about the role of ITNs in preventing malaria will have a positive impact on reducing the prevalence of malaria. In the present study, we observed some people who used Insecticide-treated nets for different purposes, for instance, toilet shelter, window and door cover, and temporary storage of maize instead of using it at night to control mosquitoes.

In the present study, the status of malaria and home area/origin were strongly associated. The odds of malaria infection who came from highland areas and traveled outside their residence are highly susceptible^14^ and carry the parasite for long periods after repeated exposures without showing clinical signs and symptoms, which in turn is influenced by lack of knowledge, low treatment-seeking behavior and travel cost^45–47^. They become asymptomatic persistent carriers when repeatedly exposed after the first exposure, as they develop partial immunity after repeated exposures^48^. This may be because travelers are less likely to use ITNs and other protection techniques.

In this study, people with experience in outdoor activities at the night were more likely to have malaria infection than their counterparts. This is comparable with the study conducted in Sanja^39^, and Debre Elias District, Northwest Ethiopia^22^. These could be the outdoor activities at night that can expose the individuals to mosquito bites more often.

Malaria infection was significantly associated with a family history of malaria. This is consistent with a study conducted in Debre Elias District^22^, Northwest Ethiopia. The risk of malaria infections was higher in those individuals who had a family history of malaria. This might be due to family members with a history of malaria infection may become a reservoir for *Plasmodium* parasite and sources of infection for the rest of the family members.

## Limitations

The study was unable to diagnose all participants and all *Plasmodium* species by nested PCR due to limited resources because of Covid-19. This study was conducted in a minor malaria transmission period that could underestimate the prevalence and does not address the major transmission season. Furthermore, the lack of significant occurrences of malaria among individuals who use ITNs compared to those who do not may be due to the fact that non-ITN users could have utilized alternative methods of prevention, thus masking any potential differences in effectiveness.

## Conclusions

The finding of this study indicates that asymptomatic malaria is common among farmworkers in Upper Awash Agro-industry. Sleeping outside/outdoor activities at night, the family history of malaria, and the absence of frequent exposure to malaria place migrant farmworkers at a very significant risk of developing malaria in the study area. The use of highly sensitive molecular diagnostics in large epidemiological studies offers a more accurate assessment of the magnitude of asymptomatic infections. Thus, the treatment of asymptomatic carriers is vital, and persistent malaria prevention tools and control strategies, particularly focusing on migrant farmworkers, should be deliberated to accomplish successful malaria control and elimination efforts in the study area. Additional studies are required for a better understanding of *Plasmodium* species infections and their contribution to the dynamics of malaria transmission and the incidence of asymptomatic infections.

## Data Availability

Data is provided within the manuscript.

## References

[1] WHO (2003). Malaria Entomology and Vector Control.

[2] Roughton S, Green A (2012). Plasmodium knowlesi malaria: Assessing the risk to the British Armed Forces. J. R. Army Med. Corps.

[3] WHO. World malaria report 2020: 20 years of global progress and challenges (2020).

[4] Lindblade KA, Steinhardt L, Samuels A, Kachur SP, Slutsker L (2013). The silent threat: Asymptomatic parasitemia and malaria transmission. Expert Rev. Anti Infect. Ther..

[5] Laishram DD, Sutton PL, Nanda N, Sharma VL, Sobti RC, Carlton JM (2012). The complexities of malaria disease manifestations with a focus on asymptomatic malaria. Malar. J..

[6] Nigatu A, Homa G, Getachew D, Gelaw S, Andersson S, Subramaniam S (2014). Can training Health Extension Workers in the integrated pharmaceutical logistics system (IPLS) be effective, affordable, and opportunistic. Eth. Med. J. Ethiop. Med. J..

[7] Makanga M (2014). A review of the effects of artemether-lumefantrine on gametocyte carriage and disease transmission. Malar. J..

[8] Kun JF, Missinou MA, Lell B, Sovric M, Knoop H, Bojowald B (2002). New emerging Plasmodium falciparum genotypes in children during the transition phase from asymptomatic parasitemia to malaria. Am. J. Trop. Med. Hyg..

[9] Stevenson JC, Stresman GH, Gitonga CW, Gillig J, Owaga C, Marube E (2013). Reliability of school surveys in estimating geographic variation in malaria transmission in the western Kenyan highlands. PLoS ONE.

[10] WHO (2012). Scaling Up Diagnostic Testing, Treatment and Surveillance for Malaria.

[11] Adhanom T, Deressa W, Witten K, Getachew A, Seboxa T, Berhane Y, Haile-Mariam D, Kloos H (2006). Malaria. Epidemiology and Ecology of Health and Disease in Ethiopia.

[12] Schicker RS, Hiruy N, Melak B, Gelaye W, Bezabih B, Stephenson R (2015). A venue-based survey of malaria, anemia and mobility patterns among migrant farm workers in Amhara Region, Ethiopia. PLoS ONE.

[13] Central Statistical Agency of Ethiopia (2013). Population projection of Ethiopia for all Regions at Wereda Level from 2014–2017.

[14] Aschale Y, Mengist A, Bitew A, Kassie B, Talie A (2018). Prevalence of malaria and associated risk factors among asymptomatic migrant laborers in West Armachiho District, Northwest Ethiopia. Res. Rep. Trop. Med..

[15] WHO (2010). Basic Malaria Microscopy.

[16] Nwagha UI, Ugwu VO, Nwagha TU, Anyaehie BU (2009). Asymptomatic Plasmodium parasitaemia in pregnant Nigerian women: Almost a decade after roll back malaria. Trans. R. Soc. Trop. Med. Hyg..

[17] Johnston SP, Pieniazek NJ, Xayavong MV, Slemenda SB, Wilkins PP, da Silva AJ (2006). PCR as a confirmatory technique for laboratory diagnosis of malaria. J. Clin. Microbiol..

[18] Pinheirob VE, Thaithongc S, Browna KN (1993). High sensitivity of detection of human malaria parasites by the use of nested polymerase chain reaction. Mol. Biochem. Parasitol..

[19] Snounou G, Singh B (2002). Nested PCR Analysis of Plasmodium Parasites.

[20] Ouédraogo AL, Bousema T, Schneider P, De Vlas SJ, Ilboudo-Sanogo E, Cuzin-Ouattara N (2009). Substantial contribution of submicroscopical Plasmodium falciparum gametocyte carriage to the infectious reservoir in an area of seasonal transmission. PloS one.

[21] Graves PM, Richards FO, Ngondi J, Emerson PM, Shargie EB, Endeshaw T (2009). Individual, household and environmental risk factors for malaria infection in Amhara, Oromia and SNNP regions of Ethiopia. Trans. R. Soc. Trop. Med. Hyg..

[22] Abebaw A (2019). The Prevalence of Symptomatic and Asymptomatic Malaria and Its Associated Factors in Debre Elias District Communities.

[23] Golassa L, Baliraine FN, Enweji N, Erko B, Swedberg G, Aseffa A (2015). Microscopic and molecular evidence of the presence of asymptomatic Plasmodium falciparum and Plasmodium vivax infections in an area with low, seasonal and unstable malaria transmission in Ethiopia. BMC Infect. Dis..

[24] Karlekar S, Deshpande M, Andrew R (2012). Prevalence of asymptomatic Plasmodium vivax and Plasmodium falciparum infections in tribal population of a village in Gadchiroli district of Maharashtra state, India. An. Int. J..

[25] Minwuyelet A, Eshetu T, Milikit D, Aschale Y (2020). Prevalence and risk factors of asymptomatic plasmodium infection in Gondar Zuria District, Northwest Ethiopia. Infect. Drug Resist..

[26] Molla E, Ayele B (2015). Prevalence of malaria and associated factors in Dilla town and the surrounding rural areas, Gedeo Zone, Southern Ethiopia. J. Bacteriol. Parasitol..

[27] Sumari D, Mwingira F, Selemani M, Mugasa J, Mugittu K, Gwakisa P (2017). Malaria prevalence in asymptomatic and symptomatic children in Kiwangwa, Bagamoyo district, Tanzania. Malar. J..

[28] Chourasia M, Raghavendra K, Bhatt R, Swain D, Valecha N, Kleinschmidt I (2017). Burden of asymptomatic malaria among a tribal population in a forested village of central India: A hidden challenge for malaria control in India. Public Health.

[29] Dal-Bianco MP, Köster KB, Kombila UD, Kun JF, Grobusch MP, Ngoma GM (2007). High prevalence of asymptomatic *Plasmodium falciparum* infection in Gabonese adults. Am. J. Trop. Med. Hyg..

[30] Baliraine FN, Afrane YA, Amenya DA, Bonizzoni M, Menge DM, Zhou G (2009). High prevalence of asymptomatic Plasmodium falciparum infections in a highland area of western Kenya: A cohort study. J. Infect. Dis..

[31] Mabunda S, Aponte JJ, Tiago A, Alonso P (2009). A country-wide malaria survey in Mozambique. II. Malaria attributable proportion of fever and establishment of malaria case definition in children across different epidemiological settings. Malar. J..

[32] Vafa M, Troye-Blomberg M, Anchang J, Garcia A, Migot-Nabias F (2008). Multiplicity of *Plasmodium falciparum* infection in asymptomatic children in Senegal: Relation to transmission, age and erythrocyte variants. Malar. J..

[33] Dunyo S, Milligan P, Edwards T, Sutherland C, Targett G, Pinder M (2006). Gametocytaemia after drug treatment of asymptomatic *Plasmodium falciparum*. PLOS Clin. Trial..

[34] Abossie A, Bekele A, Yohanes T, Abera A (2017). Prevalence of asymptomatic *Plasmodium falciparium* and *Plasmodium vivax* malaria carriage among school children of malaria endemic areas of Mirab Abaya district, Southern Ethiopia. J. Parasitol. Vector Biol..

[35] Huang F, Takala-Harrison S, Liu H, Xu J-W, Yang H-L, Adams M (2017). Prevalence of clinical and subclinical *Plasmodium falciparum* and Plasmodium vivax malaria in two remote rural communities on the Myanmar–China Border. Am. J. Trop. Med. Hyg..

[36] Zaw MT, Thant M, Hlaing TM, Aung NZ, Thu M, Phumchuea K (2017). Asymptomatic and sub-microscopic malaria infection in Kayah State, eastern Myanmar. Malar. J..

[37] Shannon KL, Khan WA, Sack DA, Alam MS, Ahmed S, Prue CS (2016). Subclinical *Plasmodium falciparum* infections act as year-round reservoir for malaria in the hypoendemic Chittagong Hill districts of Bangladesh. Int. J. Infect. Dis..

[38] Beyene H, Telele N, Mekuria A (2015). Asymptomatic malaria and associated factors in Pawe, Northern Ethiopia. Int. J. Infect. Dis. Trop. Med..

[39] Worku L, Damtie D, Endris M, Getie S, Aemero M (2014). Asymptomatic malaria and associated risk factors among school children in Sanja town, Northwest Ethiopia. Int. Schol. Res. Not..

[40] Haji Y, Fogarty AW, Deressa W (2016). Prevalence and associated factors of malaria among febrile children in Ethiopia: A cross-sectional health facility-based study. Acta Trop..

[41] Tadesse F, Fogarty AW, Deressa W (2018). Prevalence and associated risk factors of malaria among adults in East Shewa Zone of Oromia Regional State, Ethiopia: A cross-sectional study. BMC Public Health.

[42] Golassa L, Baliraine FN, Enweji N, Erko B, Swedberg G, Aseffa A (2015). Microscopic and molecular evidence of the presence of asymptomatic *Plasmodium falciparum* and *Plasmodium vivax* infections in an area with low, seasonal and unstable malaria transmission in Ethiopia. BMC Infect. Dis..

[43] Eke R, Chigbu L, Nwachukwu W (2006). High prevalence of asymptomatic Plasmodium infection in a suburb of Aba Town, Nigeria. Anna. Afr. Med..

[44] Mahajan B, Zheng H, Pham PT, Sedegah MY, Majam VF, Akolkar N (2012). Polymerase chain reaction–based tests for pan-species and species-specific detection of human Plasmodium parasites. Transfusion.

[45] Gryseels C, Grietens KP, Dierickx S, Xuan XN, Uk S, Bannister-Tyrrell M (2015). High mobility and low use of malaria preventive measures among the Jarai male youth along the Cambodia–Vietnam border. Am. J. Trop. Med. Hyg..

[46] Alemu K, Worku A, Berhane Y, Kumie A (2014). Men traveling away from home are more likely to bring malaria into high altitude villages, northwest Ethiopia. PLoS One.

[47] Alemu A, Tsegaye W, Golassa L, Abebe G (2011). Urban malaria and associated risk factors in Jimma town, south-west Ethiopia. Malar. J..

[48] Doolan DL, Dobaño C, Baird JK (2009). Acquired immunity to malaria. Clin. Microbiol. Rev..

